# Supplementary material for: Analysis of chi angle distributions in free amino acids via multiplet fitting of proton scalar couplings
Source: Magn Reson (Gott). 2024 Aug 19;5(2):103–20. doi: 10.5194/mr-5-103-2024 (PMC11570886; doi:10.5194/mr-5-103-2024)
Supplement: The supplement related to this article is available online at: https://doi.org/10.5194/mr-5-103-2024-supplement. [file mr-5-103-2024-supplement.zip › Syed2024/data/fit1d_fitnmr_output.tar.gz!/fit1d/pro/500/bmse000047_simulation_1_nmredata/1H/resonances_1d.pdf]

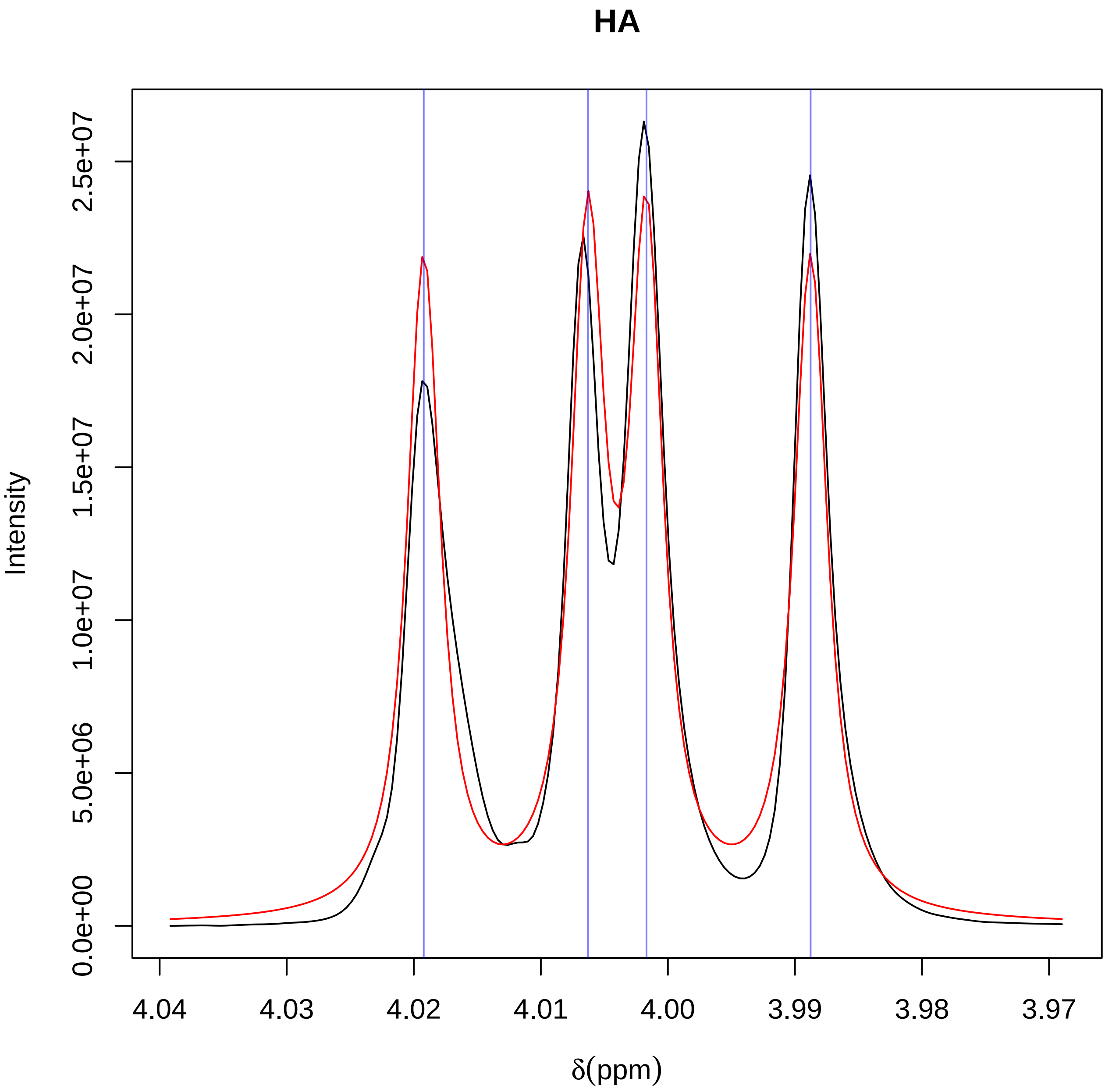
HA
Intensity
0.0e+00
5.0e+06
1.0e+07
1.5e+07
2.0e+07
2.5e+07
4.04
4.03
4.02
4.01
4.00
3.99
3.98
3.97
δ(ppm)

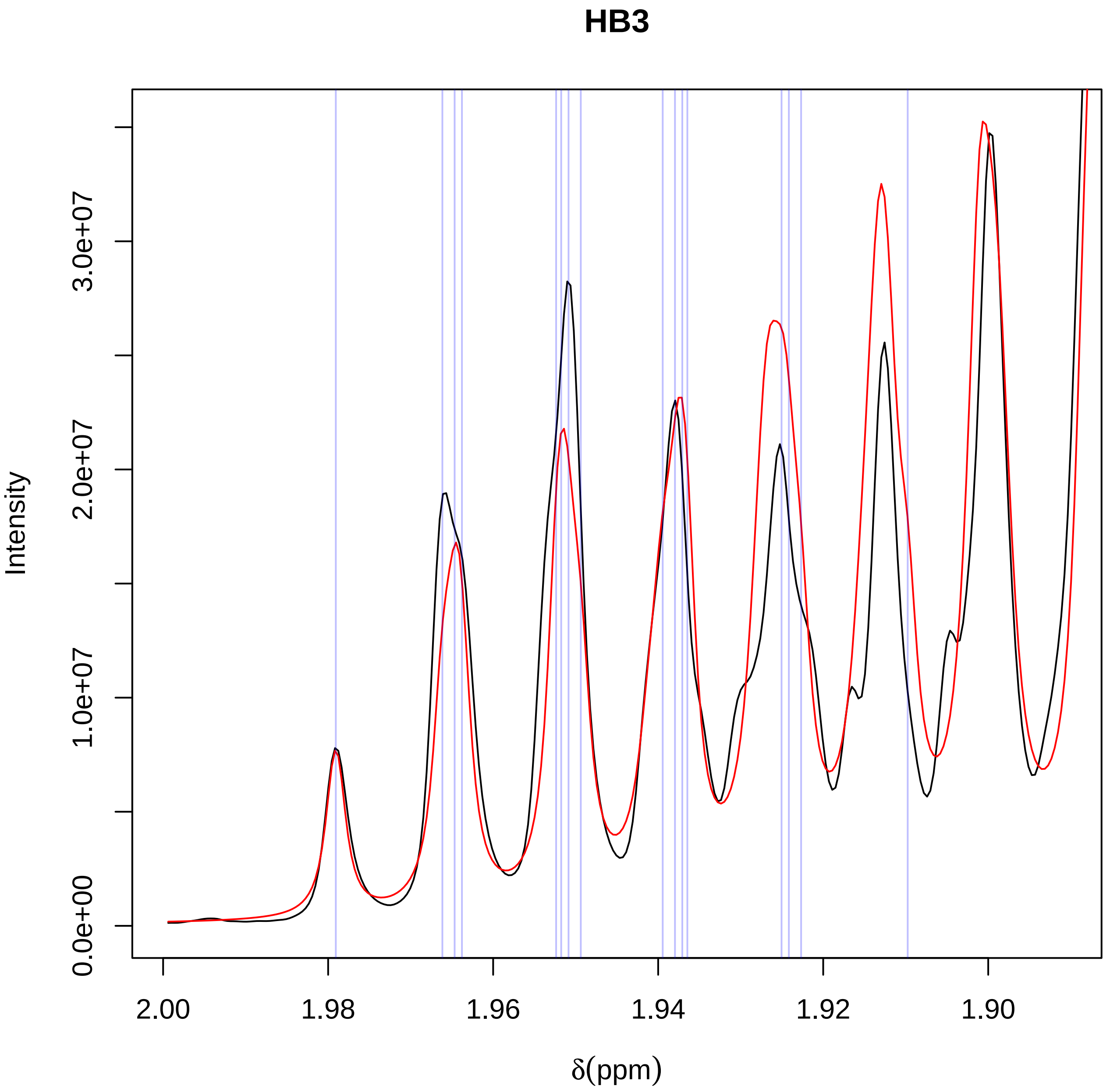

HB3
Intensity
δ(ppm)
0.0e+00
1.0e+07
2.0e+07
3.0e+07
2.00
1.98
1.96
1.94
1.92
1.90

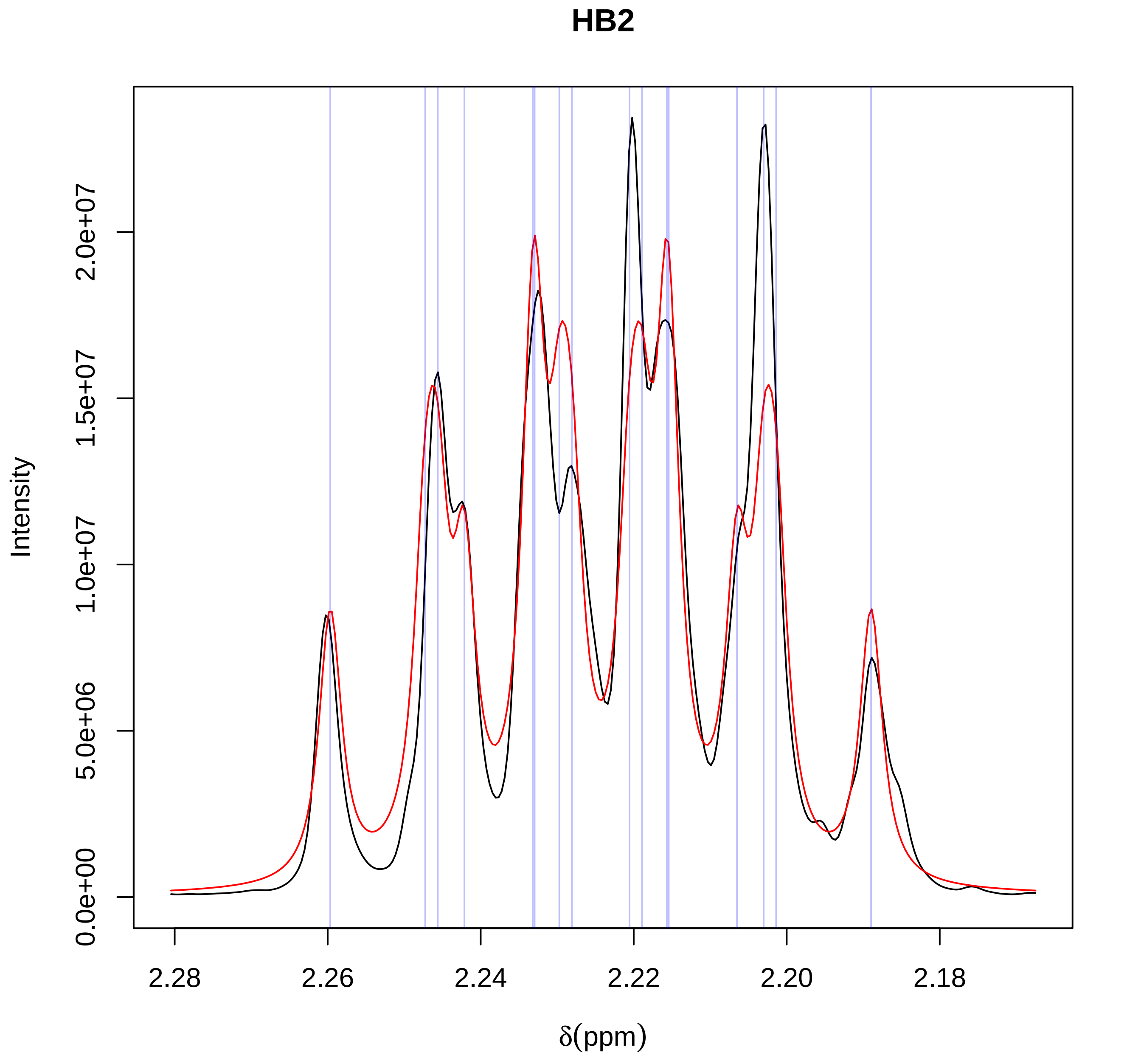

HB2
Intensity
2.0e+07
1.5e+07
1.0e+07
5.0e+06
0.0e+00
2.28
2.26
2.24
2.22
2.20
2.18
δ(ppm)

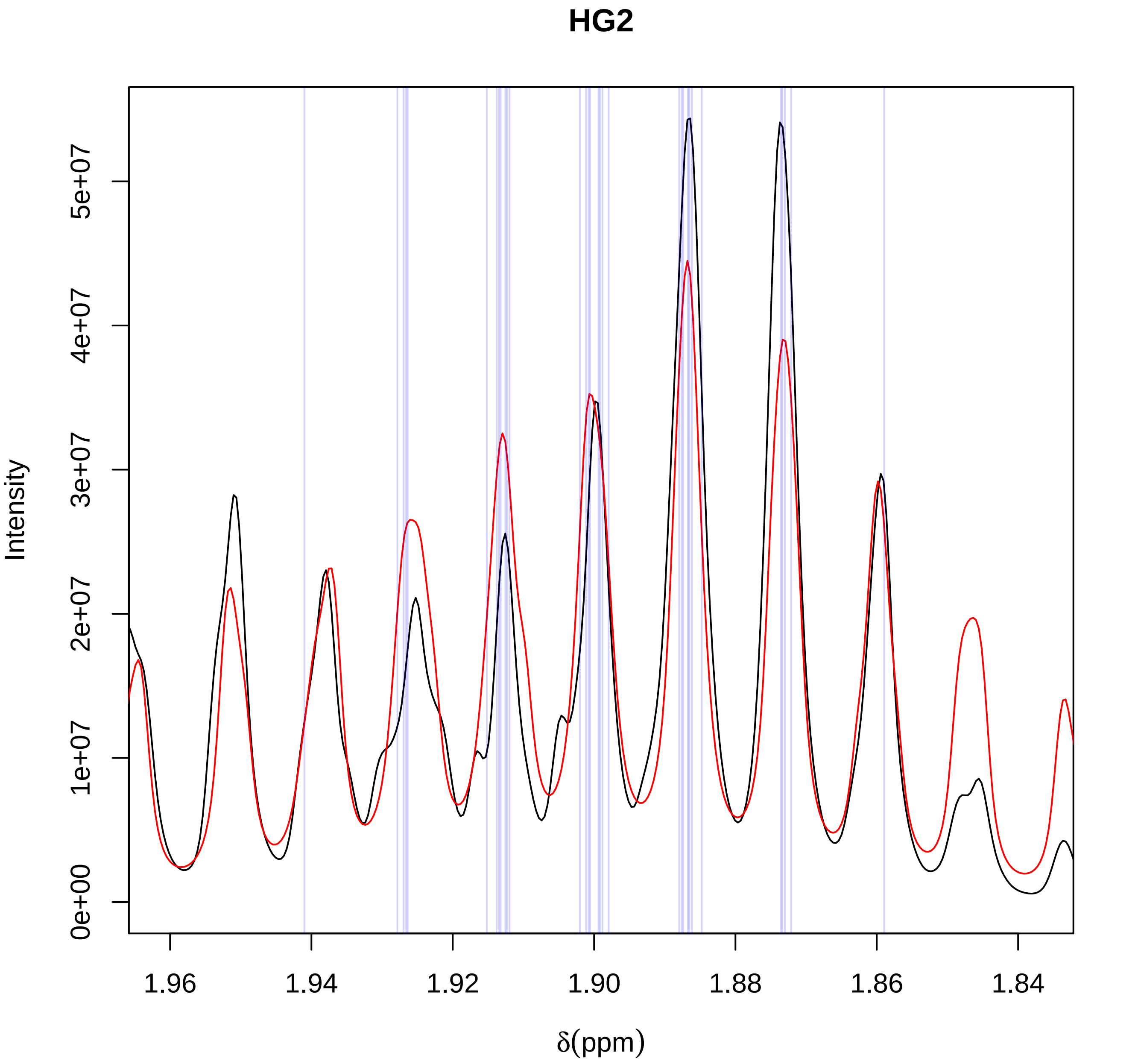

HG2
Intensity
δ(ppm)
0e+00
1e+07
2e+07
3e+07
4e+07
5e+07
1.96
1.94
1.92
1.90
1.88
1.86
1.84

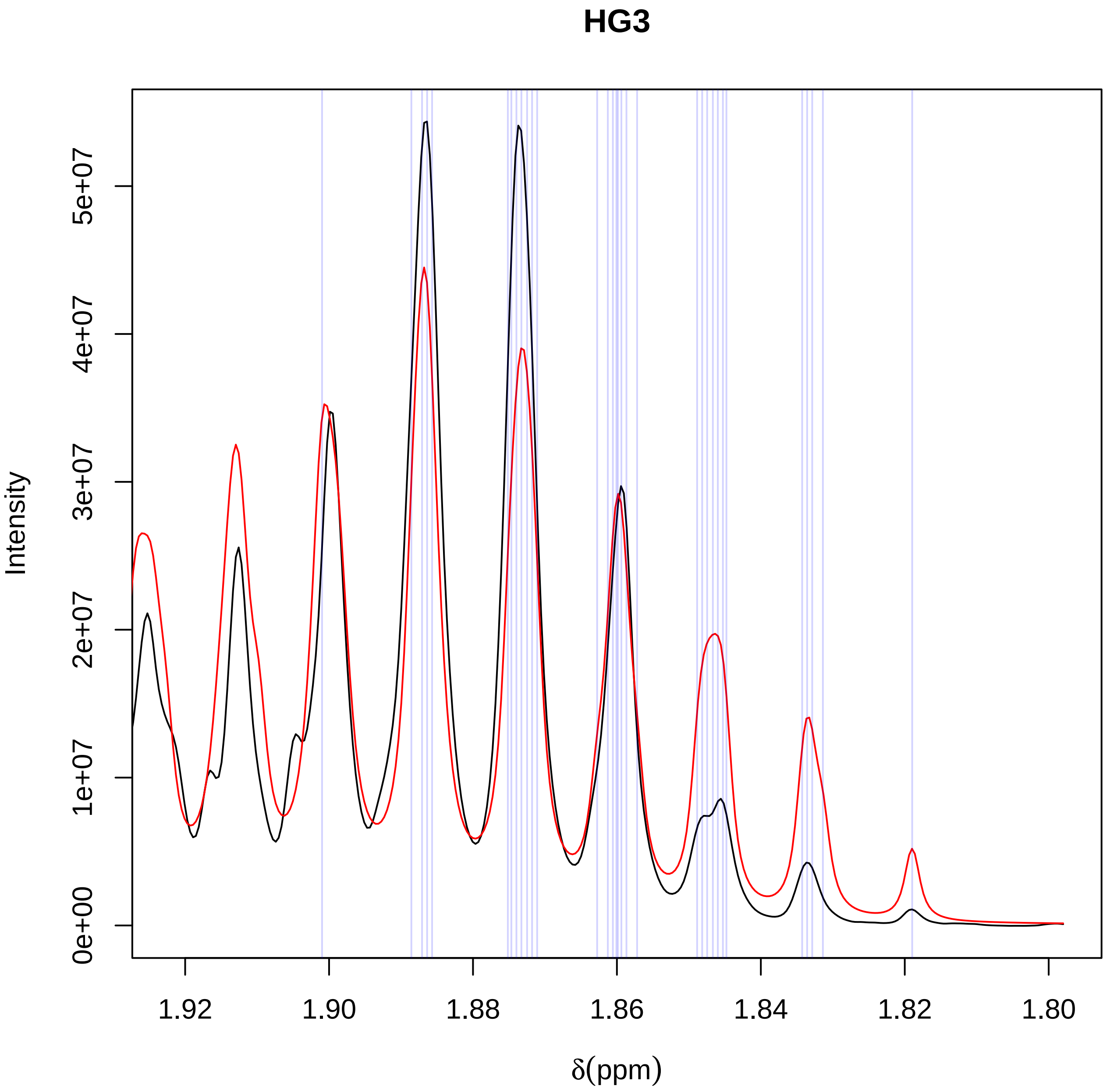

HG3
Intensity
δ(ppm)
0e+00
1e+07
2e+07
3e+07
4e+07
5e+07
1.92
1.90
1.88
1.86
1.84
1.82
1.80

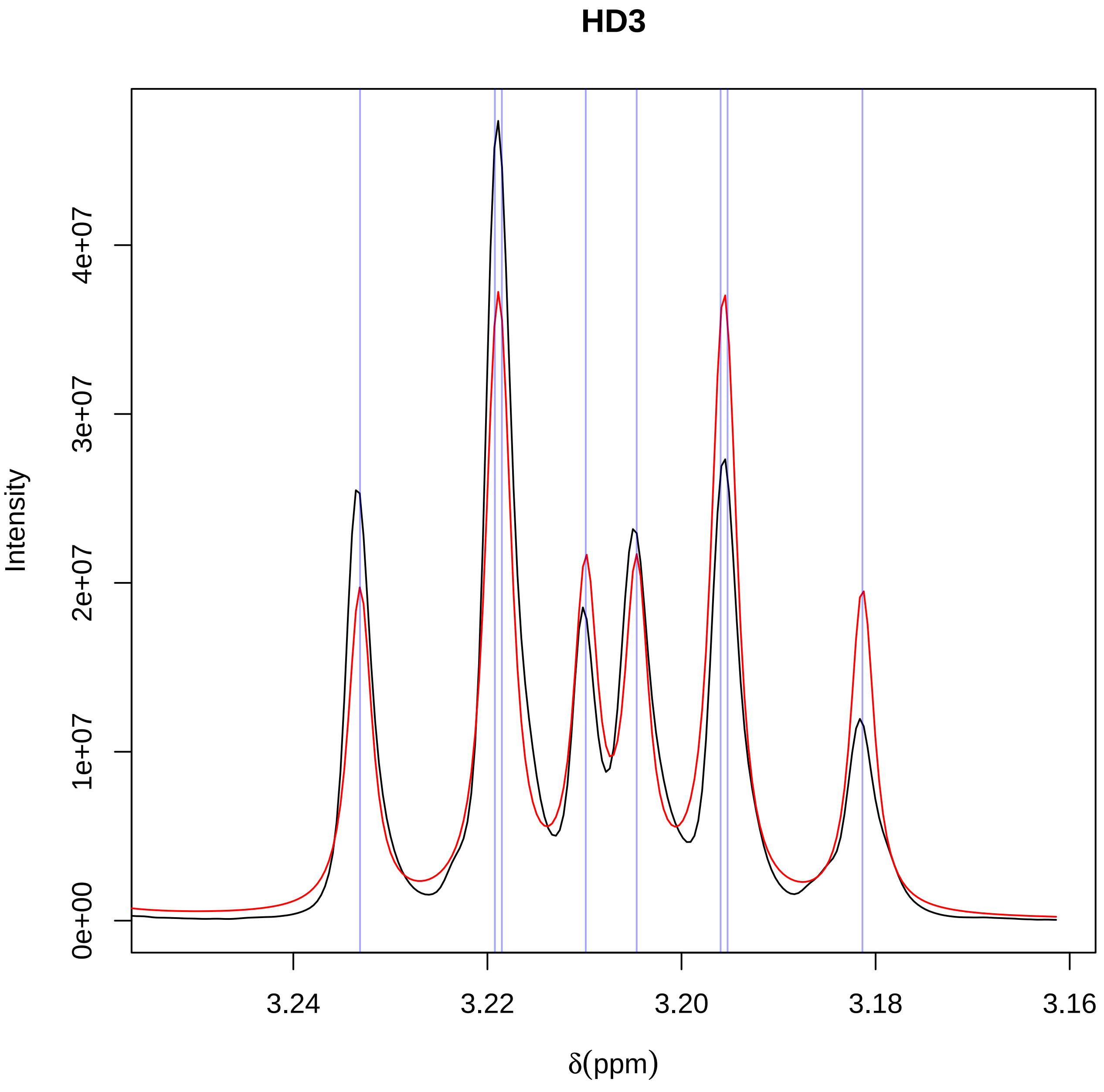
HD3
Intensity
0e+00
1e+07
2e+07
3e+07
4e+07
3.24
3.22
3.20
3.18
3.16
δ(ppm)

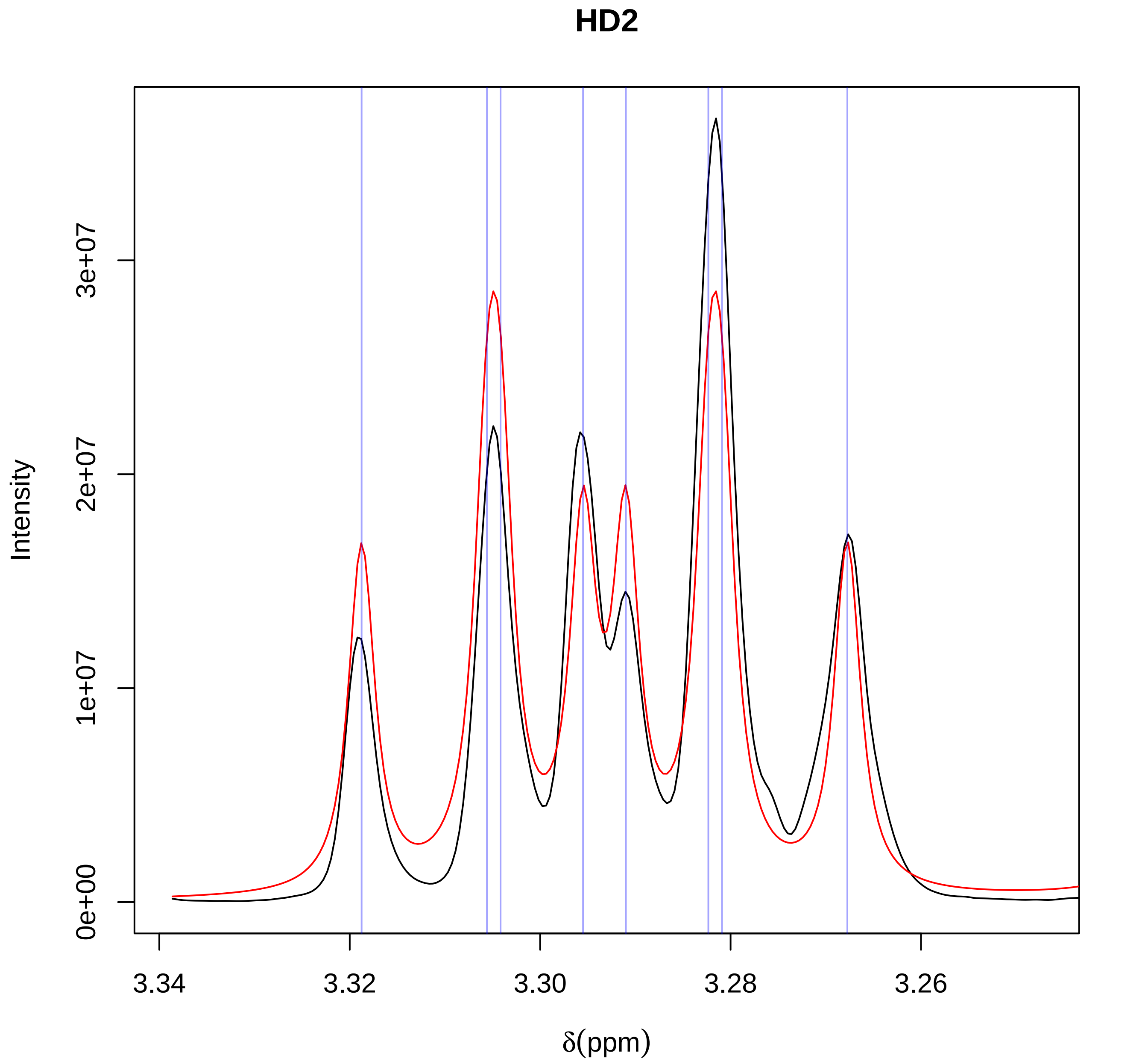
HD2
Intensity
0e+00
1e+07
2e+07
3e+07
3.34
3.32
3.30
3.28
3.26
δ(ppm)
